# A Comprehensive Clinico-Pathological Analysis of Osseous Neoplasms: An Observational Study in a Tertiary Care Facility

**DOI:** 10.7759/cureus.71937

**Published:** 2024-10-20

**Authors:** Meenakshi Mohapatro, Rajesh Rana, Pratiksha Mishra, Kalyani Gouda, Lipsa Priyadarshini, Priyadarshini Swain, Lity Mohanty

**Affiliations:** 1 Pathology, Srirama Chandra Bhanj Medical College and Hospital, Cuttack, IND; 2 Orthopedics, Srirama Chandra Bhanj Medical College and Hospital, Cuttack, IND; 3 Orthopedics, Institute of Medical Sciences and Sum Hospital, Bhubaneswar, IND

**Keywords:** bone neoplasm, histopathological evaluation, malignancies, osseous neoplasm, tumors

## Abstract

Introduction: Bone neoplasms represent a diverse group of malignancies that significantly impact morbidity and mortality. These tumors primarily affect the appendicular skeleton and, while less common than other malignancies, are particularly notable due to their prevalence in adolescents and young adults. Given their potential for rapid growth and high metastatic potential, these neoplasms can be life-threatening. This study aims to explore the relative frequencies, age, and sex distributions, as well as the anatomical and clinico-pathological characteristics of bone tumors in a tertiary care hospital.

Materials and methods: This retrospective cross-sectional study was conducted in the histopathology section of the Department of Pathology at a tertiary care medical college in Western India over 18 months (January 2023 to June 2024). All histopathologically confirmed cases of bone tumors, both benign and malignant, were analyzed based on the WHO's recent fifth-edition classification. Clinical data, including age, sex, and anatomical site, along with radiological investigations, were also reviewed for final diagnosis.

Results: A total of 63 patients, ranging from three months to 66 years old, with a mean age of 25.8 years, were included in the study. Of these, 34 (53.9%) were male and 29 (46.03%) were female, resulting in a male-to-female ratio of 1.17:1. The highest incidence of primary bone tumors occurred in the 11-20 years age group. Benign tumors accounted for 36 cases (57.11%), while 27 cases (42.85%) were malignant. Giant cell tumors (GCTs) were the most common, with 12 cases (19.04%), followed by osteochondroma with 11 cases (17.46%). Among malignant tumors, osteosarcoma was the most frequent, with 11 cases (17.46%), followed by Ewing’s sarcoma with 10 cases (15.87%).

Conclusion: The majority of tumors in this cohort were benign, with a slight male predominance, particularly among young adults. The diagnosis of bone tumors requires a comprehensive approach, including clinical evaluation, radiological correlation, and histopathological examination (HPE). In certain cases, immunohistochemistry is essential for an accurate diagnosis, staging, and the development of an effective treatment plan.

## Introduction

Osseous neoplasms are relatively uncommon when compared to other neoplasms in our body. It comprises of 0.5% of the total world cancer incidence according to the global incidence of bone tumors [1]. Primary osseous neoplasms constitute 0.2% of the total malignancies reported worldwide [2]. In the current WHO 2020 classification, osseous neoplasms have been categorized based on their tissue of origin constituting both benign and malignant counterparts. Benign osseous neoplasms are more common but they often go undetected due to their asymptomatic nature and, hence, possess difficulties in diagnosis [3]. The overall incidence of osseous neoplasms presents with a bimodal pattern, the majority of which is seen during adolescence and in individuals over 60 years [4]. Etiologically, most bone tumors may arise de novo or may arise in association with benign precursor lesions or diseased bone as in Paget’s disease, prior chemotherapy, irradiation, and less commonly due to chronic infections and trauma [5].

With the aim to understand the different patterns of osseous neoplasms including their relative frequencies, age and sex distributions, and clinico-pathological presentation of the various tumors this study was carried out in a tertiary care facility over a period of 18 months. This study highlights the importance of histopathological examination (HPE) in conjunction with clinico-radiological features to arrive at a final diagnosis.

## Materials and methods

Study design and setting 

A retrospective cross-sectional study was carried out in the Department of Pathology of a tertiary care hospital in eastern India over 18 months (from January 2023 to June 2024).

Data collection

Complete history, clinico-radiological details, and preoperative investigation findings, wherever necessary, were collected and analyzed for all cases.

Sample processing

Biopsy samples, irrespective of the technique used, were fixed in 10% buffered neutral formalin overnight. Biopsies containing bony tissue were kept in 5%-10% nitric acid for two to three days. The decalcified tissue was processed using increasing concentrations of alcohol, and paraffin-embedded blocks were made. Thin sections of 4-6μ were cut using a microtome and stained with hematoxylin and eosin (H&E) for HPE.

Classification of osseous neoplasms

Osseous neoplasms were classified according to the latest WHO 2020 classification of bone tumors, which includes osteogenic tumors (e.g., osteosarcoma and osteoma), chondrogenic tumors (e.g., chondrosarcoma and chondroma), fibrogenic tumors (e.g., fibrosarcoma and fibroma), Ewing sarcoma family tumors, giant cell tumors (GCTs), notochordal tumors (e.g., chordoma), vascular tumors (e.g., hemangioendothelioma), and other malignant bone tumors (e.g., adamantinoma and undifferentiated pleomorphic sarcoma) [6].

Radiological data collection and data analysis

Radiological investigations were collected for final comparison and analysis from the case records in the Department of Radiology. Data tabulation and analysis were conducted to determine the relative frequency, age, and gender distributions, presented as numbers or percentages.

Inclusion and exclusion criteria

A total of 63 clinically and histopathologically proven osseous neoplasms, comprising both benign and malignant counterparts, were included in this study. Non-neoplastic lesions of bone were excluded from the study.

## Results

Gender distribution of osseous neoplasms

The gender distribution of osseous neoplasms in our study revealed a higher prevalence in males, with 34 cases (53.9%), compared to 29 cases (46.03%) in females. This corresponds to a male-to-female ratio of 1.17:1, indicating a slightly higher occurrence of osseous neoplasms in males within the studied cohort (Table 1).

**Table 1:** Gender distribution of cases

Gender	Number of cases	%
Male	34	53.9
Female	29	46.03
Total	63	100

Age distribution of osseous neoplasms

The age distribution analysis showed that the highest number of cases occurred in the 11-20 years age group, accounting for 23 cases (36.5%). Both the <10 years and 31-40 years age groups reported seven cases each (11.11%). The lowest incidence was observed in individuals over 60 years of age, with only two cases (3.17%). This data suggests a higher prevalence of osseous neoplasms among adolescents and young adults (Table 2).

**Table 2:** Age-wise distribution of cases

Age group (years)	Number of cases	%
0-10	7	11.11
11-20	23	36.5
21-30	12	19.04
31-40	7	11.11
41-50	8	12.69
51-60	3	4.76
61-70	2	3.17
Total	63	100

Behavioral categorization of osseous neoplasms

In terms of tumor behavior, 36 out of 63 cases (57.11%) were benign, while 27 cases (42.85%) were malignant. This indicates that benign osseous neoplasms were more frequent in our study population than malignant ones (Table 3).

**Table 3:** Osseous neoplasms based on behavior

Behavior of lesions	Number of cases	%
Benign	36	57.11
Malignant	27	42.85
Total	63	100

Tissue of origin according to WHO classification

**Figure 1:**
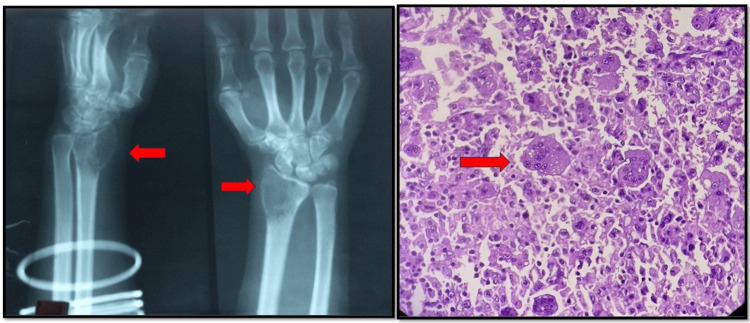
X-ray and histopathology of GCT of distal radius X-ray of distal radius shows a GCT imparting a typical soap bubble appearance with thinning of the cortex, and HPE shows numerous osteoclast-like giant cells in between mononuclear neoplastic cells, characterized by round to oval shapes with pale eosinophilic cytoplasm, pleomorphic nuclei, evenly dispersed chromatin, and prominent nucleoli. HPE: histopathological examination; GCT: giant cell tumor

**Figure 2:**
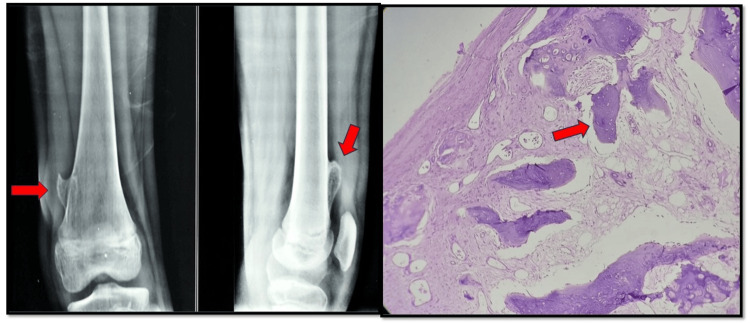
Osteochondroma distal femur Osteochondroma involves the distal portion of the femur, and H&E (100×) shows a fibrous capsule, underlying cartilaginous cap with underlying bony trabeculae. H&E: hematoxylin and eosin

**Figure 3:**
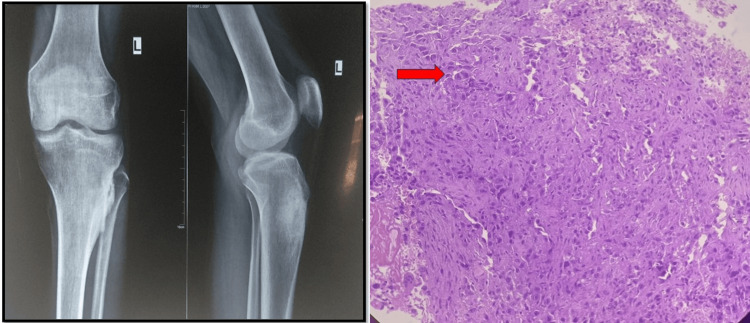
Radiograph and histopathology showing osteosarcoma of proximal tibia X-ray shows expansile osteosarcoma of the proximal tibia, and HPE shows markedly pleomorphic tumor cells surrounding the native trabeculae, with a few atypical mitoses seen. HPE: histopathological examination

**Figure 4:**
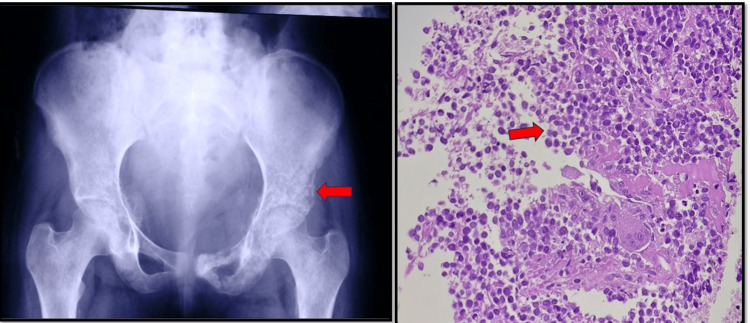
Ewing sarcoma of ilium X-ray shows an osteolytic permeative lesion involving the iliac bone. H&E (100x): HPE shows small round cells in between the normal bony trabeculae, with a few tumor giant cells seen. HPE: histopathological examination; H&E: hematoxylin and eosin

When categorized according to the latest WHO classification of bone tumors, tumors of cartilaginous and osteoclast-rich origin were the most prevalent, each contributing 19 cases to the total. Among benign neoplasms, GCTs were the most common, with 12 cases (19.04%), followed by osteochondroma with 11 cases (17.46%). Among malignant neoplasms, osteosarcoma was the most frequently observed, with 11 cases (17.46%), closely followed by Ewing’s sarcoma with 10 cases (15.87%) (Table 4). (Figures 1, 2, 3, 4).

**Table 4:** Bone tumors according to the tissue of origin (benign and malignant) GCT: giant cell tumor

Osseous neoplasms	Number of cases	%
Chondrogenic tumors		
Enchondroma	4	6.34
Osteochondroma	11	17.46
Chondromyxoid fibroma	1	1.58
Chondrosarcoma	3	4.76
Osteogenic tumors		
Osteosarcoma	11	17.46
Fibrogenic tumors	-	-
Vascular tumors	-	-
Osteoclastic giant cell-rich tumors		
Aneurysmal bone cyst	7	11.11
GCT of bone	12	19.04
Notochordal tumors	-	-
Other mesenchymal tumors		
Fibrous dysplasia	1	1.58
Hematopoietic neoplasms of bone		
Solitary plasmacytoma of bone	1	1.58
Langerhans cell histiocytosis	2	2.98
Ewing's sarcoma	10	15.87
Total	63	100

Incidence of individual tumors by age and gender

The incidence of individual osseous tumors was further analyzed by age group and gender, according to the latest WHO classification of bone tumors. This detailed categorization provides insights into the demographic and biological variations in the presentation of osseous neoplasms within the study population (Table 5).

**Table 5:** Incidence of bone tumors based on age and gender GCT: giant cell tumor

Osseous neoplasm	<20 yrs	20-40 yrs	>40 yrs	Males %	Females %
Enchondroma	1	-	3	25	75
Osteochondroma	7	1	3	72.7	27.2
Chondromyxoid fibroma	-	1	-	100	-
Chondrosarcoma	-	1	2	33.3	66.67
Osteosarcoma	8	2	1	60	40
Fibrogenic tumors	-	-	-	-	-
Vascular tumors	-	-	-	-	-
Aneurysmal bone cyst	4	2	1	71.42	28.57
GCT of bone	1	8	3	41.66	58.33
Notochordal tumors	-	-	-	-	-
Fibrous dysplasia	1	-	-	-	100
Solitary plasmacytoma of bone	-	-	1	100	-
Langerhans cell histiocytosis	2	-	-	100	-
Ewing's sarcoma	5	4	1	40	60
Total	29	19	15	-	-

## Discussion

This was a retrospective cross-sectional study carried out to interpret the spectrum of various osseous neoplasms in a tertiary care facility and gives us an idea about the relative frequencies, age, and gender distribution. We have also compared and contrasted our data to existing literature on osseous neoplasms. Our review shows that the age of presentation is similar to other studies conducted on osseous neoplasms [5]. On histopathological diagnosis, out of a total of 63 lesions, among neoplastic lesions, the incidence of benign tumors was 36 cases (57.11%), and malignant tumors were 27 (42.85 %) shown in Table 3, which is comparable with the studies conducted by Gururajprasad C et al. (Table 6) and Yopovinu Rhutso et al. [7,8].

**Table 6:** Comparison of our study with other Indian studies M: male; F: female

Osseous neoplasms	Gururajaprasad C et al.	Patel et al.	Modi et al.	Present study
Gender predominance	M:F=2.65:1	M:F=1.5:1	M>F	M:F=1.17:1
Peak age groups	2^nd^-3^rd^ decades	11-30yrs	25-50 yrs	11-20 years
Benign neoplasms	56.25%	Non-neoplastic: 41.25%, benign lesions: 27.5%	Non-neoplastic: 74.5%, benign lesions: 17.64%	57.11%
Malignant neoplasms	43.75%	20%	7.8	42.85%

Among the benign tumors, the most common benign tumor was a GCT (Figure 1) in 12 cases (19.04%) as shown in Table 4. The study conducted by Naz et al. and Settakom et al. also shows GCT as the most common benign lesion while the study conducted by Kannan et al. shows osteochondroma as the most common benign tumor [9-11]. This is in contrast to most Asian as well as Western literature, in which osteochondroma is the most common benign tumor and GCT is the second most common [12]. GCT patients were mostly 20-40 years of age with a female predominance, in agreement with the study conducted by Mohammad et al. [13].

The other benign tumors following osteochondroma (Figure 2) were aneurysmal bone cysts and enchondroma with seven and four cases, respectively. ABC shows a predominance in young adults and males, whereas enchondroma shows a predominance in the elderly with female predominance in our setting. Osteosarcoma was a common malignant tumor (Figure 3), with 11 cases (17.46%) seen in all other studies, especially by Bahebeck et al., shown in Table 4 [14].

The peak age incidence of primary bone tumors was in the age group of 11-20 years, which is in accordance with a study conducted by Abdulkareem FB et al. [12]. It showed a male predominance similar to other studies [11]. Most tumors of the bone showed male preponderance with male to female ratio of 1.17:1 similar to other studies as shown in Table 1.

Ewing sarcoma is a highly malignant, undifferentiated, aggressive peripheral primitive neuroectodermal tumor occurring most commonly at the diaphysis of long bones (Figure 4), seen most commonly in <20 yrs age group, with female predominance in our study [12,13].

In the present study, there were three cases of chondrosarcomas forming 4.7% of all osseous neoplasms commonly seen in the age above 60, with a female predominance. Other uncommon osseous neoplasms found in our setting were chondromyxoid fibroma (one case), fibrous dysplasia (one case), solitary plasmacytoma (one case), and Langerhans cell histiocytosis (two cases).

This study's limitations include its retrospective design and single-center setting, which may affect generalizability. The small sample size and reliance on histopathology alone may have led to diagnostic challenges, particularly for rare or ambiguous tumors. Additionally, variability in diagnostic criteria across studies complicates direct comparisons with existing literature.

## Conclusions

Osseous neoplasms pose a great diagnostic challenge both for the clinician and the pathologists. Although most of the benign and malignant lesions can be interpreted based on the site of occurrence, few may mimic malignancy. Radiological investigations such as X-rays and MRIs also play a substantial role in guiding toward correct diagnosis based on sites of occurrence. Thus histopathological diagnosis is the gold standard for exact diagnosis with few cases requiring confirmation by immunohistochemistry so as to aid the clinician toward proper staging, providing appropriate treatment, and in prediction of the prognosis of the variety of bone neoplasms.
